# Microvesicles from Human Immortalized Cell Lines of Endothelial Progenitor Cells and Mesenchymal Stem/Stromal Cells of Adipose Tissue Origin as Carriers of Bioactive Factors Facilitating Angiogenesis

**DOI:** 10.1155/2020/1289380

**Published:** 2020-06-15

**Authors:** Agnieszka Krawczenko, Aleksandra Bielawska-Pohl, Maria Paprocka, Honorata Kraskiewicz, Agnieszka Szyposzynska, Elżbieta Wojdat, Aleksandra Klimczak

**Affiliations:** Laboratory of Biology of Stem and Neoplastic Cells, Hirszfeld Institute of Immunology and Experimental Therapy, Polish Academy of Sciences, R. Weigla 12, 53-114 Wroclaw, Poland

## Abstract

Endothelial progenitor cells (EPCs) and mesenchymal stem/stromal cells (MSCs) are associated with maintaining tissue homeostasis and tissue repair. Both types of cells contribute to tissue regeneration through the secretion of trophic factors (alone or in the form of microvesicles). This study investigated the isolation and biological properties of microvesicles (MVs) derived from human immortalized MSC line HATMSC1 of adipose tissue origin and EPC line. The human immortalized cell line derived from the adipose tissue of a patient with venous stasis was established in our laboratory using the hTERT and pSV402 plasmids. The human EPC line originating from cord blood (HEPC-CB.1) was established in our previous studies. Microvesicles were isolated through a sequence of centrifugations. Analysis of the protein content of both populations of microvesicles, using the Membrane-Based Antibody Array and Milliplex ELISA showed that isolated microvesicles transported growth factors and pro- and antiangiogenic factors. Analysis of the miRNA content of isolated microvesicles revealed the presence of proangiogenic miRNA (miR-126, miR-296, miR-378, and miR-210) and low expression of antiangiogenic miRNA (miR-221, miR-222, and miR-92a) using real-time RT-PCR with the TaqMan technique. The isolated microvesicles were assessed for their effect on the proliferation and proangiogenic properties of cells involved in tissue repair. It was shown that both HEPC-CB.1- and HATMSC1-derived microvesicles increased the proliferation of human endothelial cells of dermal origin and that this effect was dose-dependent. In contrast, microvesicles had a limited impact on the proliferation of fibroblasts and keratinocytes. Both types of microvesicles improved the proangiogenic properties of human dermal endothelial cells, and this effect was also dose-dependent, as shown in the Matrigel assay. These results confirm the hypothesis that microvesicles of HEPC-CB.1 and HATMSC1 origin carry proteins and miRNAs that support and facilitate angiogenic processes that are important for cutaneous tissue regeneration.

## 1. Introduction

The primary function of stem/progenitor cells in adult organisms is the maintenance of tissue homeostasis and repairing the tissue in which they reside [1]. Among the many types of stem/progenitor cells, mesenchymal stem/stromal cells (MSCs) are studied extensively due to their immunomodulatory properties and the ability to direct endogenous tissue repair. MSCs are undifferentiated, multipotent, nonhematopoietic cells with the ability to self-renew and differentiate and reside in different tissues and organs. Mesenchymal stem/progenitor cells can be isolated from various tissues, including bone marrow, cord blood, placenta, skin, skeletal muscles, dental pulp, and adipose tissue [2–5]. Bone marrow, umbilical cord blood, and adult peripheral blood are also common sources of endothelial progenitor cells (EPCs). These cells, first described by Asahara et al. [6], in addition to their ability to differentiate into mature endothelial cells, can secrete various proangiogenic factors, thus contributing to angiogenesis and vascular repair [7]. Both MSCs and EPCs take part in tissue regeneration by releasing a variety of growth factors, including factors with a proangiogenic capacity. Neovascularization is essential for a proper blood supply necessary to maintain tissue homeostasis and proper function in many ischemic diseases, including ischemic cardiomyopathy, ischemic stroke, ischemic limb, and chronic wounds (reviewed by Bian et al. 2019 [8]) Wound healing is a complex and dynamic process that progresses through a sequence of phases involving hemostasis, inflammation, proliferation, epithelialization, angiogenesis, remodeling, and scarring [9]. The complexity of the wound healing process is related to the activity of different types of cells, including endothelial cells, fibroblasts, keratinocytes, and immune cells [9, 10]. These cells cooperate during tissue repair, influencing each other through a variety of bioactive factors, which they secrete [11, 12]. Angiogenesis is part of the proliferative phase of wound healing, and proper revascularization of ischemic tissue warrants tissue recovery. In ischemic conditions, the secreted factors facilitate communication between injured tissue cells and cells involved in the immune response. This communication is supported by diverse types of microvesicles released by MSCs [8, 13].

In recent years, evidence has been growing that the regenerative effects of tissues are accomplished through a cooperation of several types of MSC-derived secretomes, including soluble factors and extracellular vesicles produced by almost all types of cells (for a review, see [14, 15]). One of the main groups of extracellular vesicles, in addition to the well-known exosomes, is microvesicles, i.e., vesicles derived from the plasma membrane ranging in size from 100 to 1000 nm, shed from the cell surface. The contribution of microvesicles to wound healing was studied by many research groups [16–18]. In the regeneration of the ischemic tissue, microvesicles mediate the modulation of immune interactions, anti-inflammatory processes, and angiogenesis, as they contain proteins, RNA, miRNA, and trophic factors derived from parent cells [13].

This study investigated the isolation and biological properties of microvesicles derived from human immortalized cell lines of adipose tissue-derived MSC (ATMSC) and EPC origin. We analyzed the content of cytokines and trophic factors of both populations of microvesicles, the presence of proangiogenic miRNA, and the effect of isolated microvesicles on the angiogenic properties of dermal endothelial cells. Moreover, we investigated the proliferation of cells involved in cutaneous regeneration, i.e., fibroblasts, keratinocytes, and endothelial cells in the presence of isolated microvesicles.

## 2. Materials and Methods

### 2.1. Cells

A human endothelial progenitor cell line originating from cord blood (HEPC-CB.1) and human normal skin microvascular endothelial cells (HSkMEC.2) were established and patented by our research group in cooperation with Kieda et al. from Centre National de la Recherche Scientifique, France, according to the previously described method [19, 20]. These endothelial cells were cultured in Opti-MEM with GlutaMAX (Thermo Fisher Scientific Inc., USA) supplemented with 2% fetal bovine serum (FBS, HyClone, UK) and 1% penicillin-streptomycin (Sigma-Aldrich, USA) and were routinely passaged using a 0.05% trypsin/0.02% EDTA (*w*/*v*) solution (IITE PAN, Poland).

The human immortalized cell line of adipose tissue-derived MSC HATMSC1 was established in our laboratory using the hTERT and pSV402 plasmids. Cells were obtained by the liposuction of abdominal fat from a patient with a venous ulcer and separated enzymatically in the CELUTION 800 (Cytori Therapeutics, USA) system. The protocol was approved by the local bioethics committee at the Regional Specialist Hospital, Research and Development Center in Wroclaw (No. KB/27/2015). All procedures were carried out in accordance with the accepted ethical standards contained in the Declaration of Helsinki. Proliferating cells, grown in DMEM supplemented with 10% fetal bovine serum (FBS, HyClone, UK), were transfected with the pSV3-neo plasmid carrying a complete SV40 early region of the large T antigen (http://www.addgene.org/vector-database/4267/*).* After selection with G418, the cells were retransfected with the pBABE-puro-hTERT plasmid (http://www.addgene.org/1771/) and selected with puromycin. The transfections were performed with the ViaFect™ transfection reagent (Promega, USA) in a serum-free medium, according to the manufacturer's instructions.

A HaCaT cell line was purchased from DKFZ [21]. A MSU-1.1 cell line was obtained through the v-myc oncogene transformation of foreskin fibroblasts [22]. The cells were cultured in DMEM supplemented with 10% fetal bovine serum (FBS, HyClone, UK), L-glutamine (Sigma-Aldrich, USA), and 1% penicillin-streptomycin (Sigma-Aldrich, USA) and were routinely passaged using a 0.05% trypsin/0.02% EDTA (*w*/*v*) solution (IITD PAN, Poland).

### 2.2. Flow Cytometric Characteristics of the Human Mesenchymal Stem Cell Line HATMSC1

Cells were detached using the nonenzymatic cell dissociation solution (Sigma Aldrich, USA) and labeled with PE-conjugated antibodies specific for human CD73, CD90, CD105, HLA ABC, HLA DR, and with FITC-conjugated antibody for CD45; and with the appropriate isotypic controls for 30 min at 4°C. After washing with PBS, the cells were analyzed using a FACSCalibur flow cytometer (Becton Dickinson, USA), and the data were processed using the CellQuest software (BD Biosciences, USA). The histograms were created using the WinMDI 2.8 Program.

### 2.3. Microvesicle Preparation and Identification

Cells were cultured in multilayer cell culture flasks (Nunc TripleFlasks, Thermo Fisher Scientific Inc.) until they reached 75% of confluence in dedicated culture media. Next, the cells were exposed to hypoxic conditions (1% of oxygen) to mimic the hypoxic environment of ischemic tissue and to enhance the release of microvesicles in serum-free media for 48 h. The culture medium was collected into centrifuge tubes, and microvesicles were isolated through a sequence of centrifugations (10 min × 300 g, 10 min × 2000 g, and 30 min × 12000 g, 4°C). Next, the microvesicles were washed in PBS (30 min × 12000 g) and stored in PBS at -80°C. Microvesicles obtained from 9–13 preparations were pooled for further experiments (see below). The homogeneity and purity of the pooled microvesicles were verified using dynamic light scattering (DLS, Malvern Zetasizer, UK), while the number of microvesicles was calculated using flow cytometry and fluorescent counting beads (CountBright™ Absolute Counting Beads for flow cytometry, Molecular Probes). Additionally, the phenotype of isolated microvesicles was evaluated by flow cytometry. Microvesicles derived from HEPC-CB.1 cells were labeled with PE-conjugated antibodies for human CD133 and CD271 antigens, which are markers of HEPC-CB.1 cells. Microvesicles derived from HATMSC1 cells were labeled with PE-conjugated antibodies for human CD73, CD90, and CD105 antigens, which are markers specific for MSC. The appropriate isotypic control was used. Microvesicles were labeled for 30 min at 4°C and were analyzed using a BD LSRFortessa flow cytometer (Becton Dickinson, USA). The data were processed using the FACSDiva™ software (BD Biosciences, USA). The histograms were created using the Flowing Software 2 program.

For functional tests, different ratios of the number of microvesicles per cell were investigated. The most optimal ratio was found to be 100 : 1 (100 microvesicles per 1 cell) based on a preliminary assay performed on the ratios 10 : 1, 25 : 1, 50 : 1, 75 : 1, and 100 : 1. Therefore, the ratios of 10 : 1 and 100 : 1 were chosen for further experiments.

### 2.4. Microvesicle Internalization

HSkMEC.2, MSU-1.1, and HaCaT cells were seeded into a 24-well plate at a density of 25 × 10^3^ cells per well and cultured in standard conditions for 24 h. Fluorescence staining of microvesicles was performed using a Vybrant Multicolor Cell Labeling Kit (Thermo Fisher Scientific Inc., USA). The microvesicles (from both the HEPC-CB.1 and HATMSC1 cell lines) were resuspended in 100 *μ*L PBS, labeled with 0.5 *μ*L of a green fluorescent reagent (DiO) and incubated at 37°C for 5 min according to the manufacturer's recommendations. After washing with PBS, the microvesicles were resuspended in a DMEM supplemented with 10% FBS and added to the cells at a ratio of 5 : 1 (5 microvesicles per cell). The ratio 5 : 1 was chosen for better visualization of internalization process, as higher ratio resulted in poor resolution. The internalization of microvesicles into the target cells was analyzed after 24 h using an Axio Observer Inverted Microscope (Zeiss, Germany) equipped with a dry 40x objective. The EGFP filter set was used to detect the labeled microvesicles. Image acquisition and processing were performed with the Zen Blue software (Zeiss, Germany).

After 24 h of incubation with microvesicles, the cells were detached using a trypsin/EDTA solution, washed once with PBS, and analyzed with flow cytometry using FACSCalibur (Becton Dickinson, USA). The cells were detected using the FL1 channel (480 nm). The histograms were created using the Flowing Software 2 program.

### 2.5. Examination of Microvesicles for Cytokine and Trophic Factor Content

The protein content of cells and microvesicles from the HATMSC1 and HEPC-CB.1 cultures was examined using the Membrane-Based Antibody Array (Human Angiogenesis Array C1000, RayBiotech). Isolated microvesicles and cells were lysed in RIPA buffer with protein inhibitor cocktail for 10 min on ice, sonicated for 15 min, then suspended in PBS, and incubated on a protein membrane according to the manufacturer's instructions. Briefly, 2 mL of blocking buffer was applied on the membrane and incubated 30 min at room temperature. Then, 1.2 mL of HEPC-CB.1 and HATMSC1 cells and cell-derived microvesicles was incubated with a membrane overnight at 4°C. Following a series of washes, a biotinylated antibody cocktail was applied on the membrane and incubated for 2 h at room temperature. Unbound antibody was removed by series of washes, and the membrane was placed in HRP-streptavidin and incubated for 2 h at room temperature. Following a third series of washes, chemiluminescence detection was performed and bound proteins were visualized using X-ray film. A comparison of signal intensities was performed using ImageJ software (MosaicJ, Philippe Thevenaz) where relative differences in expression levels of each analyzed sample were measured and normalized to the intensities of positive control using the Protein Array Analyzer plugin. Automatic analysis of obtained data was calculated using Microsoft® Excel-based Analysis Software Tool for Human Angiogenesis kit. The results were calculated as a percentage of expression, where positive control was set to 100%, and negative control was set to 0% (relative expression). The cutoff line was set to 5%. All results above 5% were considered real expression.

To confirm semiquantitative results of the cytokines' presence in the microvesicles derived from HATMSC1 and HEPC-CB.1 cells presented on the heat map, several cytokines such as EGF, FGF-2, GRO, IL-6, IL-8, MCP-1, and RANTES were also analyzed using Human Cytokine/Chemokine Magnetic Bead Panel Milliplex® MAP Kit (Merck, Darmstadt, Germany), according to the manufacturer's instructions. Commercial standards included in the kit and samples were assessed in duplicate. Briefly, lysed microvesicles were incubated with beads overnight with shaking at 4°C (18 h, 750 rpm) on the assay plate and then washed using a hand-held magnetic block. Detection antibody was then added to each well and incubated for 1 h at room temperature. Data were acquired on a validated and calibrated MAGPIX® system (Luminex) with xPONENT® software. The median fluorescence intensity (MFI) of standards, control, and samples was measured and analyzed in Milliplex Analyst software using a five-parameter logistic curve-fitting method for calculating cytokine concentrations in samples.

### 2.6. Examination of MicroRNAs Present in Microvesicles

The presence of four angiomiRs (miR-126, miR-296, miR-378, and miR-210) and three antiangiomiRs (miR-221, miR-222, and miR-92a) in microvesicles was investigated using real-time RT-PCR with the TaqMan technique. Total cellular RNA was isolated from 5 × 10^6^ of HEPC-CB.1 and HATMSC1 microvesicles using the NucleoSpin RNA kit (MACHEREY-NAGEL, Germany). First-strand cDNA was synthesized through a reverse transcription of 10 ng of total RNA using the TaqMan MicroRNA Reverse Transcription Kit (Thermo Fisher Scientific Inc., USA). The reverse transcription was carried out with microRNA primers for angiomiRs: miR-210 (assay ID 000512), miR-126 (assay ID 002228), miR-296 (assay ID 002101), and miR-378 (assay ID 001314), and for antiangiomiRs: miR-221 (assay ID 000524), miR-222 (assay ID 002276), miR-92a (assay ID 000431), and RNU48 as an internal control (assay ID 001006). Real-time PCR was then performed with the abovementioned microRNA primers in the ViiA7 Real-Time PCR System according to the TaqMan Small RNA Assays Protocol. The expression of the analyzed miRs in microvesicles was calculated relative to the controls (HEPC-CB.1 and HATMSC1 cells).

### 2.7. Proliferation Assay

Cell proliferation was investigated using the standard MTT test (Sigma-Aldrich) which measures the metabolic activity of the cells and is an indirect test to measure cell proliferation. Briefly, 3 × 10^3^ cells (HSkMEC.2, MSU-1.1, and HaCaT cell lines) were seeded onto a 96-well plate in triplicate. After adhering, the medium was changed for DMEM without serum, and microvesicles derived from the HEPC-CB.1 and HATMSC1 cells were added in two different ratios: 10 microvesicles per 1 cell (10 : 1) or 100 microvesicles per 1 cell (100 : 1). The cells growing in DMEM without serum were used as a control. The test was carried out for up to seven days without changing the medium.

### 2.8. Angiogenic Assay

Angiogenic properties of microvesicles were examined in the Matrigel (Matrigel GFR, Corning) angiogenic assay using the human microvascular endothelial cell line of dermal origin HSkMEC.2. Endothelial cells (1.5 × 10^4^/well) were seeded in duplicate onto Matrigel-coated 96-well plates in DMEM without serum and in the presence of isolated microvesicles at the 10 : 1 and 100 : 1 ratios and cultured in standard conditions for up to 24 h. The cells growing on Matrigel in DMEM without serum and without microvesicles served as a control. After 24 hours, images were acquired with a digital camera, which was part of an Olympus CKX41 microscope.

### 2.9. Statistical Analysis

Statistical analysis was performed using the GraphPad Prism 7 software. For proliferation results, two-way ANOVA and Kruskal-Wallis with Dunn's multiple comparison tests were used. Angiogenic assays were calculated using the ImageJ Angiogenesis Analyzer software.

## 3. Results

### 3.1. HATMSC1 Cell Line Characteristics

**Figure 1:**
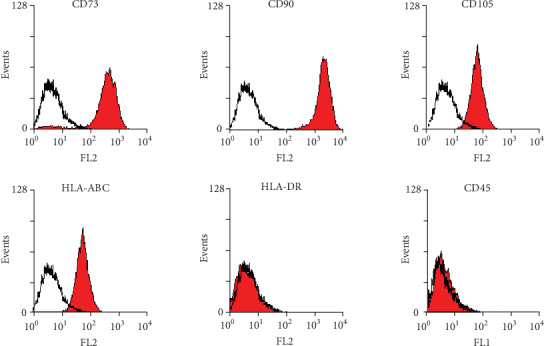
Representative analysis of the phenotype of HATMSC1 cells evaluated with flow cytometry. Cells were stained with selected antibodies conjugated with defined fluorochromes. Data are presented as a histogram overlay. Red-filled histograms correspond to MSCs labeled with defined antibodies, and unfilled histograms represent isotype controls.

The human immortalized cell line HATMSC1 was analyzed using flow cytometry for the presence of the common MSC surface antigens CD73, CD90, and CD105 and for the expression of the CD45 hematopoietic marker and HLA ABC and HLA DR antigens. The analysis confirmed the presence of CD73, CD90, and CD105 molecules on the HATMSC1 cells. The cells were also positive for HLA class I antigens, HLA ABC, but they did not express HLA class II antigens, HLA DR, and were negative for the hematopoietic marker CD45 (Figure 1).

### 3.2. Isolation of Microvesicles from HEPC-CB.1 and HATMSC1 Cell Lines

**Figure 2:**
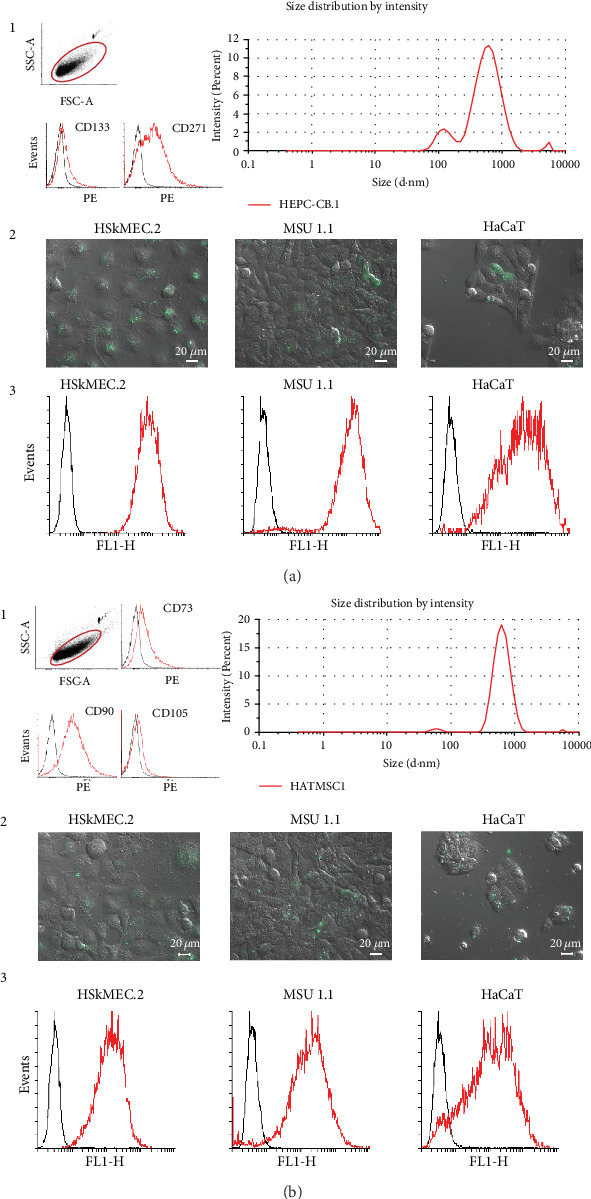
Characterization and internalization of microvesicles isolated from the EPC line (HEPC-CB.1 cell line (a)) and from adipose tissue HATMSC1 cell line (b). (a1 and b1) Isolated microvesicles were counted using fluorescent counting beads (left panel). Microvesicles were then assessed for the purity of isolation using dynamic light scattering (right panel). (a2 and b2) Representative images of microvesicle internalization into target cells: dermal microvascular endothelial cells HSkMEC.2, fibroblasts MSU 1.1, and keratinocytes HaCaT. Images were taken using an inverted microscope after 24 h of incubation with microvesicles dyed with the green fluorescent DiO dye (scale bar: 20 *μ*m). (a3 and b3) Flow cytometric histograms showing green fluorescence of recipient cells after microvesicle internalization. Black histograms represent the control for untreated cells, and red histograms represent cells treated with labeled microvesicles.

Analysis of microvesicle samples derived from both HEPC-CB.1 and HATMSC1 cells revealed that a population of microvesicles ranging in size from 100 to 1000 nm had been successfully isolated using the serial centrifugation protocol. The flow cytometric analysis confirmed the presence of CD133 and CD271 antigens on microvesicles isolated from HEPC-CB.1 cells. Moreover, the CD73, CD90, and CD105 molecules were detected on the HATMSC1-derived microvesicles (Figure 2, a1 and b1). Dynamic light scattering was used to compare the size distribution and purity of the isolated microvesicles. Using this technique, the average size of HEPC-CB.1-derived microvesicles was evaluated at 447.3 nm and the average size of HATMSC1-derived microvesicles was evaluated as 584.6 nm. In all subsequent experiments, a pooled fraction of microvesicles from at least nine different isolations was used.

### 3.3. Microvesicle Internalization

Microvesicles derived from both HEPC-CB.1 and HATMSC1 cells, incubated with target cells for 24 h, were incorporated into skin-derived cell lines, including vessel endothelial cells HSkMEC.2, fibroblasts MSU-1.1, and keratinocytes HaCaT, as documented by the presence of microvesicles showing green fluorescence in the cytoplasm of the examined cells (Figure 2, a2 and b2). The internalization of the isolated microvesicles into the target cells was additionally confirmed by cytometric analysis (Figure 2, a3 and b3).

### 3.4. Cytokine, Trophic Factor, and miRNA Content of Microvesicles

**Figure 3:**
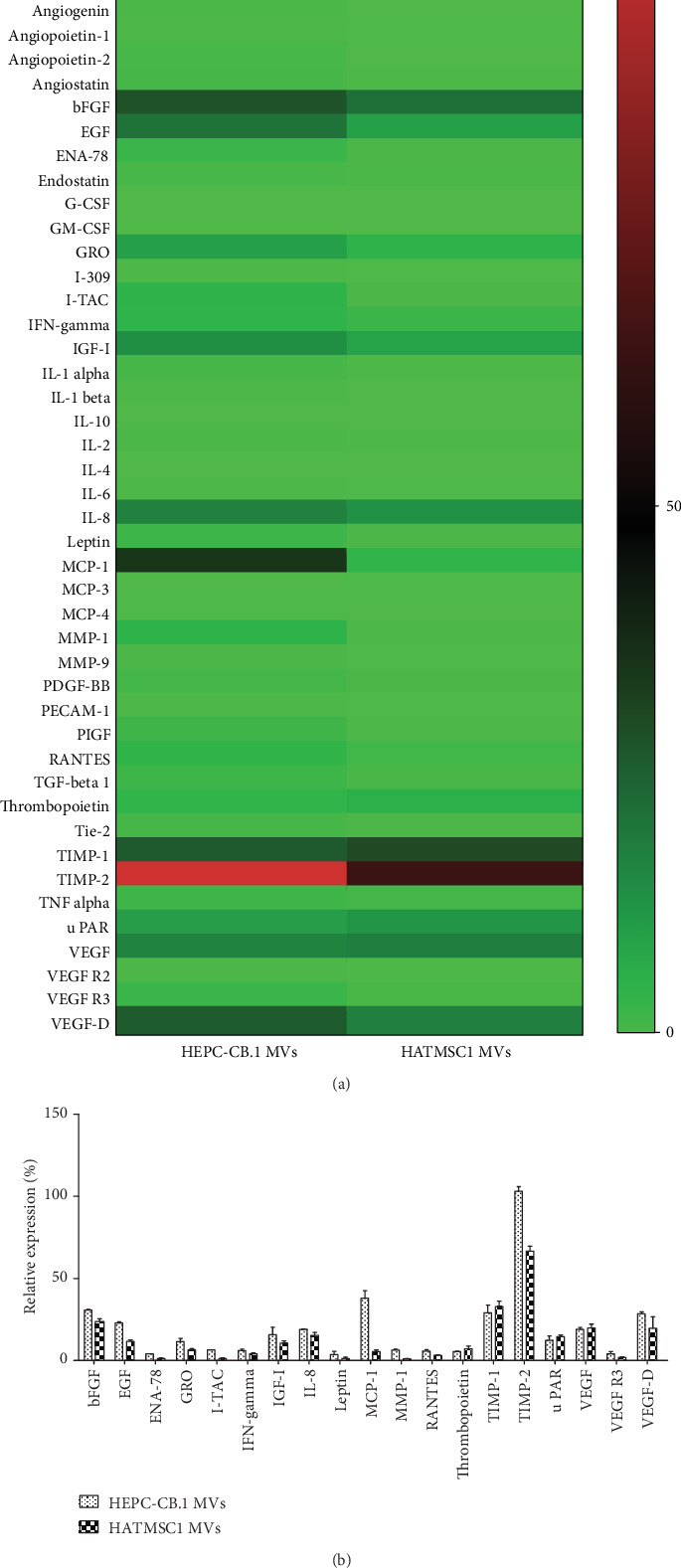
Analysis of cytokine and trophic factor content of microvesicles (MVs) derived from the EPC line (HEPC-CB.1) and adipose tissue HATMSC1 cell line. Analysis was performed using the Membrane-Based Antibody Array (RayBiotech). (a) A heat map was created using the GraphPad Prism 7 software; (b) the column graph presents selected proteins (equal/above 5% of positive control). Data were presented as mean ± SEM values, *n* = 2.

The content of isolated microvesicles was analyzed for the presence of cytokines, trophic factors, and microRNA. The angiogenic Membrane-Based Antibody Array was selected for protein analysis. The results comparing microvesicles derived from HEPC-CB.1 and HATMSC1 cells produced under hypoxic conditions are shown as a heat map in Figure 3(a). The HEPC-CB.1 microvesicles exhibited the expression of 19 out of 43 examined cytokines and trophic factors, whereas the HATMSC1 microvesicles showed the expression of 12 cytokines and trophic factors (relative expression equal to or above 5%, Figure 3(b)). Both HEPC-CB.1 and HATMSC1 microvesicles contained growth factors (e.g., EGF and bFGF) and pro- and antiangiogenic factors (e.g., IL-8, VEGF, TIMP-1, and TIMP-2). Additionally, HEPC-CB.1-derived microvesicles contained cytokines and molecules that regulate angiogenesis (e.g., GRO, IGF-I, I-TAC, MCP-1, MMP-1, and VEGF-D).

**Figure 4:**
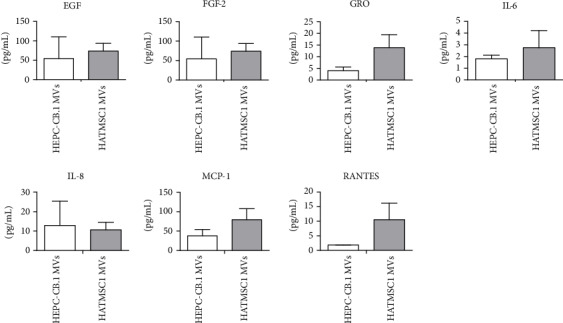
Concentrations of the selected cytokines found in HEPC-CB.1 and HATMSC1 microvesicles (MVs) measured by Milliplex ELISA. Data represents mean ± SD, *n* = 2.

The results obtained in the Membrane-Based Antibody Array were then confirmed using Milliplex ELISA assay (Figure 4). Quantitative validation of the selected factors (EGF, FGF-2, GRO, IL-6, IL-8, MCP-1, and RANTES) demonstrated that microvesicles derived from both HEPC-CB.1 and HATMSC1 cells contain a high concentration of EGF (95 pg/mL and 76 pg/mL, respectively), FGF-2 (54 pg/mL and 74 pg/mL), and MCP-1 (38 pg/mL and 79 pg/mL). In contrast, the expression level of IL-6 was only 1.8 pg/mL for HEPC-CB.1 microvesicles and 2.7 pg/mL for HATMSC1 microvesicles (Figure 4).

**Figure 5:**
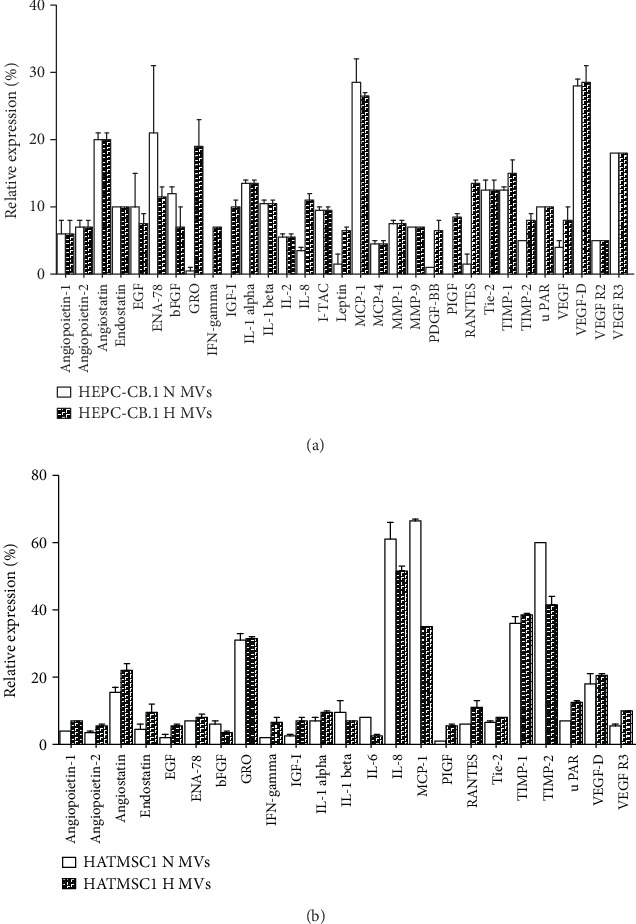
Analysis of cytokines and trophic factor content of microvesicles (MVs) derived from the HEPC-CB.1 cell line (a) and HATMSC1 cell line (b) in normoxic (N) and hypoxic (H) conditions. Analysis was performed using the Membrane-Based Antibody Array (RayBiotech). The graphs illustrate selected proteins (relative expression equal/above 5% of positive control). Data were presented as mean ± SEM values, *n* = 2.

Moreover, we compared the content of HEPC-CB.1- and HATMSC1-derived microvesicles produced both in normoxia and hypoxia (Figure 5). In hypoxia, the relative expression was augmented for 10 molecules compared to normoxic conditions in HEPC-CB.1 microvesicles (e.g., relative expression: 19% vs. 0.5% for GRO; 7% vs. 0% for IFNgamma; and 13.5% vs. 1.5% for RANTES, for hypoxia vs. normoxia, respectively). Normoxic conditions increased the relative expression for 2 molecules, ENA-78 and bFGF (21% in normoxia vs. 11.5% in hypoxia for ENA-78 and 12% in normoxia vs. 7% in hypoxia, Figure 5(a)). In HATMSC1-derived microvesicles, normoxia increased the relative expression for IL-6 (8% in normoxia vs. 2.5% in hypoxia), IL-8 (61% vs. 51.5%), MCP-1 (66.5% vs. 35%), and TIMP-2 (60% vs. 41.5%), whereas hypoxia caused augmentation in angiostatin (22% for hypoxia vs. 15.5% in normoxia) and RANTES (11% in hypoxia vs. 6% in normoxia, Figure 5(b)) expression. Hypoxic conditions had no effect on the expression of other examined proteins present in microvesicles compared to normoxia.

**Figure 6:**
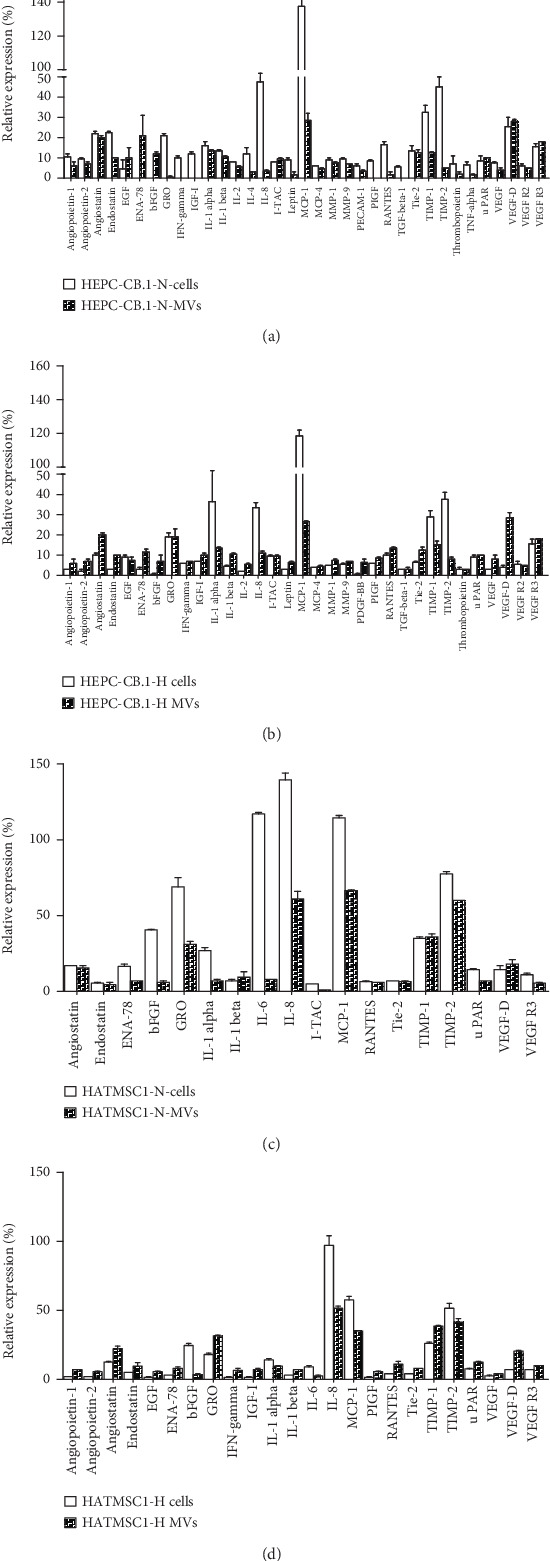
Analysis of cytokines and trophic factor content of cells and microvesicles (MVs) derived from the HEPC-CB.1 cell line (a, b) and HATMSC1 cell line (c, d) in normoxic (a, c) and hypoxic (b, d) conditions. Analysis was performed using the Membrane-Based Antibody Array (RayBiotech). The graphs present selected proteins (relative expression equal/above 5% of positive control). Data were presented as mean ± SEM values, *n* = 2.

The differences in protein expression in microvesicles and their parental cells were also tested. As shown in Figure 6, in normoxic conditions, there are a higher number of bioactive factors preferentially expressed in cells than in microvesicles (13 vs. 3 proteins in HEPC-CB.1 and 9 vs. 0 in HATMSC1 cells vs. microvesicles), whereas under hypoxic conditions, the proportions were inverted (5 vs. 7 for HEPC-CB.1 and 4 vs. 8 for HATMSC1, for cells vs. microvesicles, respectively). Interestingly, hypoxia augmented the expression of some proangiogenic factors in microvesicles, such as VEGF and VEGF-D in HEPC-CB.1 microvesicles (Figure 6(b)) and GRO and VEGF-D in HATMSC1 microvesicles (Figure 6(d)).

**Figure 7:**
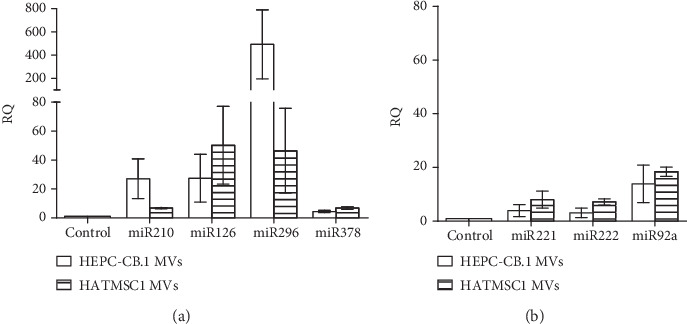
Relative expression of proangiogenic miRNAs, miR-210, miR-126, miR-296, and miR-378 (a), and antiangiogenic miRNAs, miR-221, miR22, and miR-92a (b), in microvesicles (MVs) isolated from EPCs (HEPC-CB.1 cell line) and MSCs derived from adipose tissue (HATMSC1 cell line). The expression of miRs was determined in real-time RT-PCR experiment and measured relative to the control (HEPC-CB.1 and HATMSC1 cell lines). Data were presented as mean ± SD values, *n* = 3.

It is well known that microvesicles also contain, in addition to the analyzed proteins, RNA regulatory molecules named miRNA. In this study, we investigated the expression of selected proangiogenic miRNAs present in the isolated microvesicles, i.e., miR-210, miR-296, miR-126, and miR-378, as well as antiangiogenic miRNAs: miR-221, miR-222, and miR-92a. We found that the microvesicles isolated from both HEPC-CB.1 and HATMSC1 were enriched in all examined proangiogenic miRNAs, as compared to the parental cell lines (control bars, Figure 7(a)). The highest relative expression (about 500 times, RQ = 492) was observed for miR-296 in EPC-derived microvesicles. Antiangiogenic miRNA expression was also higher in microvesicles compared to their parental cells, both HEPC-CB.1 and HATMSC1. However, the expression of antiangiogenic miRNAs was definitely lower than that of proangiogenic miRNAs; the highest relative expression observed for antiangiogenic miR-92a in HATMSC1 microvesicles was below 20 (RQ = 18) (Figure 7(b)). Although differences were observed in the level of expression of miRs between the microvesicles derived from HATMSC1 vs. microvesicles derived from HEPC-CB.1 cells, the results were not statistically significant because of a high standard deviation.

### 3.5. Effect of Microvesicles on Cell Proliferation

**Figure 8:**
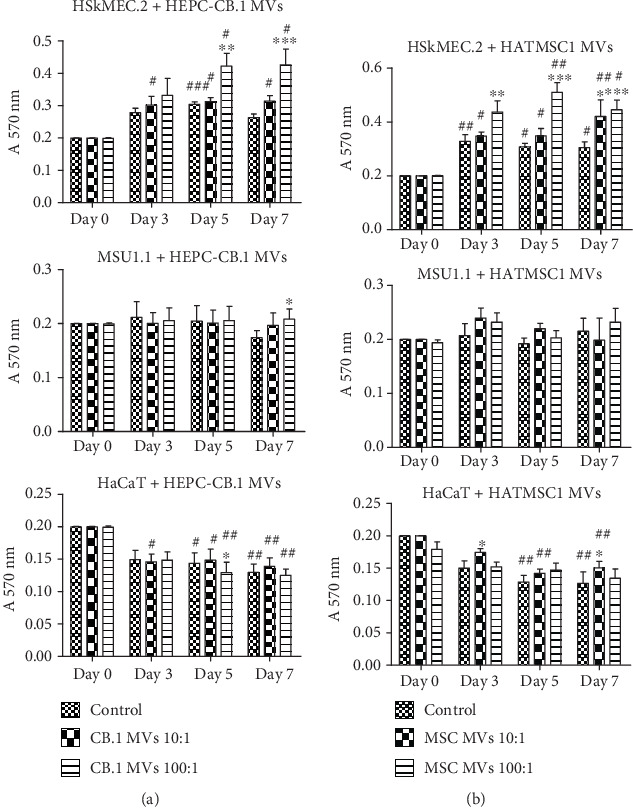
Cell proliferation evaluated with the standard MTT test. Human dermal endothelial cells HSkMEC.2, human fibroblast cell line MSU-1.1, and human keratinocyte cell line HaCaT were cultured in the presence of microvesicles (MVs) isolated from HEPC-CB.1 (a) or adipose tissue immortalized cell line HATMSC1 (b). Proliferation was assessed on days 3, 5, and 7 of culture. Results represent mean ± SEM, *n* = 6 (^∗^*p* < 0.05, ^∗∗^*p* < 0.01, and ^∗∗∗^*p* < 0.001 calculated vs. control for a defined time point; ^#^*p* < 0.05, ^##^*p* < 0.01, and ^###^*p* < 0.001 calculated at a given day vs. day 0 for each treatment).

Subsequently, the isolated microvesicles were assessed for their effect on the proliferation and angiogenic properties of the cells involved in skin regeneration. It was shown that both HEPC-CB.1- and HATMSC1-derived microvesicles increased the proliferation of human vessel endothelial cells of dermal origin, compared to the control without microvesicles on a given day, and that this effect was dose-dependent (Figure 8). The proliferation of HSkMEC.2 endothelial cells was higher when a ratio of 100 : 1 was applied (100 microvesicles per 1 cell) compared to a ratio of 10 : 1, regardless of the origin of the microvesicles. A significant increase in the proliferation of HSkMEC.2 cells was observed for the HATMSC1 microvesicles at a ratio of 100 : 1 on day 3 (*p* < 0.01), and the proliferation was maintained until day 7 (*p* < 0.001). On day 7, a significant increase in proliferation was also observed for the HATMSC1 microvesicles at a ratio of 10 : 1. The HEPC-CB.1 microvesicle treatment caused a significant increase of HSkMEC.2 cell proliferation on days 5 and 7 (*p* < 0.01 and *p* < 0.001, respectively) only at a ratio of 100 : 1. In contrast, the microvesicles, regardless of HEPC-CB.1 or HATMSC1 origin, had a limited effect on the proliferation of keratinocytes and fibroblasts (Figure 8).

However, the kinetics of proliferation during observation time was different for both HSkMEC.2 and HaCaT cells compared to day 0. The microvesicles from HEPC-CB.1 significantly increased the proliferation of HSkMEC.2 during observation time points between day 0 and 7 for both 10 : 1 and 100 : 1 ratios (*p* < 0.05). The microvesicles from HATMSC1 also increased the proliferation of HSkMEC.2 during observation time points between day 0 and 7 for both 10 : 1 (*p* < 0.01) and 100 : 1 (*p* < 0.05) ratios. In the case of the HaCaT cells treated with HATMSC1 microvesicles, proliferation decreased in all examined cell groups, including the controls, at day 3; however, the microvesicles at both 10 : 1 and 100 : 1 ratios increased HaCaT proliferation compared to the control on any given day and maintained this effect during observation time up to day 7 (Figure 8).

When comparing HaCaT cells treated with HEPC-CB.1 microvesicles, proliferation also decreased in all examined cell groups, including the controls, at day 3, similarly to treatment with HATMSC1-derived microvesicles. In the next days, the only increase of proliferation was observed for the 10 : 1 ratio on day 5 and day 7, whereas the ratio of 100 : 1 caused a decrease in proliferation rate as compared to the control.

Microvesicles originated from HEPC-CB.1 cell line did not have an influence on the proliferative potential of fibroblast cell line MSU-1.1 during observation time. HATMSC1-derived microvesicles in both concentrations 10 : 1 and 100 : 1 increased proliferative capacity of MSU-1.1; however, the effect was transient and not statistically significant.

### 3.6. Stimulation of Angiogenesis by Microvesicles

**Figure 9:**
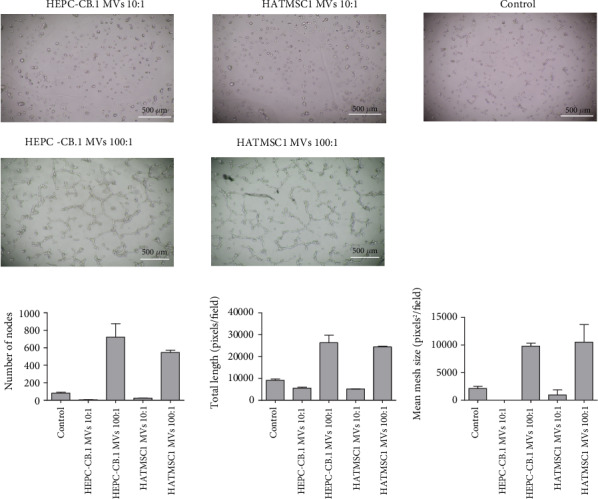
Effect of microvesicles (MVs) on pseudovessel formation on a Matrigel matrix. HSkMEC.2 dermal endothelial cells were incubated for 24 h in a medium without serum (control) or in the presence of microvesicles from EPCs (HEPC-CB.1) or adipose tissue immortalized cell line HATMSC1 at a ratio of 10 : 1 and 100 : 1. Images were captured using an Olympus CKX41 microscope, magnification 40x, and a representative example is shown. Number of nodes, total length, and mean mesh size were calculated using the ImageJ software. Results represent mean ± SD, *n* = 2.

A similar effect was observed in the angiogenic assay. As Figure 6 illustrates, microvesicles improved the angiogenic properties of endothelial cells depending on the dose. A pseudotubule formation was only visible for the ratio of 100 : 1 for the microvesicles derived from both HEPC-CB.1 and HATMSC1. In contrast, angiogenesis in the Matrigel assay was ineffective when the ratio of microvesicles to the examined cells was 10 : 1. An analysis of angiogenic parameters, such as the number of nodes, total length, and mean mesh size, revealed the same trend. The results depended on the number of microvesicles per endothelial cell. At the ratio of 100 : 1, all three parameters were higher than at the ratio of 10 : 1 and higher than in the control (Figure 9).

## 4. Discussion

Mesenchymal stem/progenitor cells of different tissue origins are considered to be a potential solution for regenerative medicine, including cutaneous healing, as they release a variety of bioactive factors that affect tissue repair and have anti-inflammatory and immunomodulatory properties [3]. In addition to the secreted proteins and trophic factors, MSCs also release small extracellular vesicles, i.e., microvesicles, into the injured microenvironment, which improve tissue regeneration. This phenomenon was also observed for EPC-derived extracellular vesicles (microvesicles and exosomes), which accelerate cutaneous wound healing by promoting angiogenesis [18, 23, 24]. Microvesicles are produced by a number of cells and are isolated from almost all organs and body fluids. Stem and progenitor cells are one of the most interesting sources of these small vesicles because microvesicles constitute the cargo of bioactive factors produced by the MSCs from which they originate [15, 16, 25]. There are several methods of isolating microvesicles. The classical approach is the well-established serial centrifugation protocol [26–28], which was also used in our study. This method is effective and simple and yields a distinct population of microvesicles ranging from 100 to 1000 nm, as confirmed by dynamic light scattering and flow cytometry.

Microvesicle content depends on the type of cells of origin, and microvesicles can transfer a variety of proteins, nucleic acids, and regulatory molecules [29]. In this study, we focused on the biological activity of microvesicles isolated from our well-established EPC line, HEPC-CB.1, originating from the perinatal tissue [20], and from a new cell line, HATMSC1, obtained from the MSCs originating from the adipose tissue collected from a patient with venous stasis. The aim of our cell line selection was to compare the proangiogenic potential of cells of different tissue origins and tissue maturity. We investigated whether the microvesicles from immortalized MSC line of adipose tissue origin had the same angiogenic properties as microvesicles isolated from unipotential EPC line. Moreover, HATMSC1 originating from the patient with venous stasis allowed us to assess the effect of donor health on the biological properties of microvesicles isolated from immortalized MSC line in terms of potential clinical application as autologous treatment, e.g., in patients with chronic diseases, such as diabetic wounds or autoimmune diseases. Studies on biological properties of adipose tissue-derived stromal cells (ASCs), originated from sclerodermic patients and healthy controls, documented that there were no differences in kinetics growth and multipotential activity of examined ASCs. Moreover, the study proved that local delivery of autologous ASCs exerts therapeutic effect on skin lesions in patients affected by chronic disease such as scleroderma [30]. These observations suggest that ASCs from patients with chronic disease are not biologically affected and MSCs isolated from adipose tissue from patients are able to secrete bioactive factors with proregenerative activity. However, primary MSCs have limited ability for cell divisions and may change their paracrine activity with subsequent number of passages [3] and these properties limited them for autologous use when treatment with repeated dose of MSCs is necessary, especially in chronic diseases. Therefore, MSC secretome and/or microvesicle from immortalized cell lines developed from a single patient may be an alternative therapeutic option for primary MSCs as autologous cell-free-based therapy for patients suffering with chronic disease. Our very recent study on the biological activity of HATMSC secretome from immortalized cell lines proved that most of the bioactive factors involved in angiogenesis were effectively produced by HATMSC lines, irrespective of the source of adipose tissue-derived MSCs (chronic wound patient vs. healthy donor), and promote human skin-origin cell proliferation involved in wound healing [31]. We also documented that immortalization procedure did not have an influence on the angiogenic activity of supernatant collected from HATMSC lines compared to primary MSCs of adipose tissue origin [31]. These observations can be translated to microvesicle composition since they contain the same trophic factors as parental cells and can be superior for storage without losing biological function.

In this study, we focused on molecules with a proangiogenic potential, carried by microvesicles, which are essential in tissue regeneration. Organ injury is associated with a reduced level of oxygen in the damaged tissue. Consequently, to mimic the hypoxic microenvironment, the parental cells, HEPC-CB.1 and HATMSC1, were cultured under hypoxic conditions to enhance the production of various paracrine factors involved in angiogenesis. Hypoxic preconditioning of parental cells may increase the levels of trophic and proangiogenic factors in MSC-derived microvesicles [32, 33] and, as reported, increase the production of extracellular vesicles [34]. EPC- and MSC-derived microvesicles contain proteins necessary for angiogenesis, such as VEGF, IL-8, and bFGF, and molecules that regulate angiogenesis, such as MCP-1 and TIMP. Quantitative evaluation of the selected factors by Milliplex ELISA confirmed high concentrations of IL-8, EGF, FGF-2, and MCP-1 as well as low concentration of IL-6 in isolated microvesicles. However, microvesicles from EPCs differ from the microvesicles originated from MSC immortalized cell line in the content of proangiogenic factors, as they contain more proangiogenic proteins compared to HATMSC1-derived microvesicles. It is also worth to notice that hypoxia augmented the relative expression for a higher number of bioactive factors involved in angiogenesis compared to normoxic conditions in EPC-derived microvesicles than in MSC-derived microvesicles. This result confirms the paracrine activity of EPCs in angiogenic processes [7]. The angiogenic potential of isolated microvesicles was also demonstrated by the expression of angiomiRs. MicroRNAs, such as miR-126, miR-296, and miR-378, have been described to play important roles in angiogenesis [35]. Our study proved the expression of four proangiogenic microRNAs: miR-126, miR-296, miR-378, and miR-210. Both HEPC-CB.1- and HATMSC1-derived microvesicles were enriched in all tested angiomiRs, as compared to the parental cells, HEPC-CB.1 and HATMSC1. However, the expression level, as expected, was higher in HEPC-CB.1-derived microvesicles, and the highest expression was observed for miR-296. The level of miR-296 rises during angiogenesis and regulates VEGFR2 expression in endothelial cells, thereby promoting angiogenesis [36, 37]. Our observations, based on microvesicle activity and miR-296 expression, also confirm the involvement of EPCs in angiogenic processes and suggest that vessel endothelial progenitors, as the more committed cells, are directly prepared to build a functional vasculature [6, 38]. An additional confirmation of the proangiogenic activity of isolated microvesicles is low expression of antiangiogenic miRNAs, such as miR-221, miR-222, and miR-92a. The level of expression of these molecules in microvesicles is much lower compared to that of proangiogenic miRNAs, which shifts the balance towards processes that promote angiogenesis. One should remember, however, that miR-221/miR-222 have a dual action in angiogenesis: in endothelial cells, these molecules inhibit angiogenesis, whereas in tumor cells, such as glioma cancer tissues and glioma cell lines, their augmented expression promotes angiogenesis (reviewed by [39]). In our studies, the low expression of miR-221 and miR-222 in microvesicles correlates with proangiogenic activity of these microvesicles.

It is also worth to notice that the EPC line is established from cells derived from umbilical cord blood, whereas the HATMSC1 cell line originates from the adipose tissue of an adult patient. The differences in the source of stem/progenitor cells and in the age of donor cells may reflect the differences in the potential of vessel endothelial progenitors and HATMSC1 cells and their derivate, such as microvesicles, to create a functional vasculature.

Isolated microvesicles carry proteins that may be responsible for several types of biological activity of cells contributing to angiogenesis. Our studies on the effect of HEPC-CB.1- and HATMSC1-derived microvesicles on the proliferation of skin-derived cells involved in skin regeneration showed that microvesicles predominantly affected the proliferation of the dermal endothelial cells HSkMEC.2. This process depended on the number of microvesicles added per endothelial cell, with a higher microvesicle/cell ratio resulting in a faster proliferation. The number of microvesicles used in our study was high enough to accelerate the proliferation of dermal endothelial cells HSkMEC.2. HEPC-CB.1 microvesicles maintain fibroblast proliferation at the control level, whereas HATMSC1-derived microvesicles transiently accelerate their proliferation potential. Microvesicles from both cell lines have no influence on keratinocyte proliferation; however, initial decrease of proliferation ability of keratinocytes was observed in control and cells treated with microvesicles. This observation is different to the findings of a recently published study conducted by Ren et al. [40], which showed that microvesicles isolated from adipose stem cells improved the proliferation of all examined cells of dermal origin, including keratinocytes and fibroblasts. On the other hand, our experiments used a varying number of intact microvesicles per cell, whereas Ren et al. used exact concentrations of microvesicles, which may explain the differences in the obtained results. Moreover, the microvesicle production and proliferation assay in Ren et al.'s study was performed using ultracentrifugated FBS, whereas our study used culture conditions without serum, which may explain the reduced proliferation response of the keratinocytes. The proliferation activity of MSCs depends on serum concentration, with a reduced percentage of FBS in culture media inhibiting proliferation but enhancing the immunosuppressive properties of MSCs [41]. The impact of culture conditions on the angiogenic potential of extracellular vesicles isolated from umbilical cord MSCs has been reported in studies on the biological activity of MSCs and their extracellular vesicles cultured in different types of xeno-free media [42].

Further experiments involving pseudotubule formation in angiogenic tests conducted on Matrigel confirmed the beneficial effect of microvesicles on angiogenesis. Microvesicles from both HATMSC1 and HEPC-C B.1 improved pseudotubule formation, as compared to endothelial cells without the support of microvesicles. Moreover, this effect also depended on the microvesicle/cell ratio, and again, a high ratio resulted in an effective pseudotubule formation, whereas the ratio of 10 : 1 was not effective for pseudovessel formation, with only small groups of cells forming. The supportive effect of microvesicles on the angiogenic properties of endothelial cells has also been described by Huaitong et al. [43]; however, they used microvesicles released by tumor cells.

Several studies describe the influence of microvesicles on the biological properties of endothelial cells. However, the term *microvesicles* used in these studies is often misleading and inadequate [44–46]. Many authors do not distinguish between microvesicles and exosomes; sometimes, the term *extravesicles* is also used. This leads to controversies regarding the obtained results, as experiments performed with “microvesicles” are, in fact, often carried out with exosomes or a mixed population of microvesicles and exosomes. An example is the study conducted by Deregibus et al. [46] on the effect of EPC-derived microvesicles on angiogenesis. Deregibus et al. described isolated vesicles as “microvesicles,” while in reality, these were rather exosomes isolated by ultracentrifugation at 100,000 g. It was also shown that the proangiogenic effect resulted from mRNA transfer, not proteins. Moreover, Deregibus et al. showed that microvesicles/exosomes isolated from bone marrow-derived MSC did not affect angiogenesis. This is in contrary to our results, where both HEPC-CB.1- and HATMSC1-derived microvesicles, defined by their size, improved angiogenic properties of adult endothelial cells.

Among the extracellular vesicles derived from MSCs, exosomes have been identified as a contributor promoting angiogenesis. Exosomes from adipose-derived MSCs contain proangiogenic miRNA (miR-126, miR-132), which enhances blood vessel formation through a release of trophic factors, such as VEGF, EGF, and FGF [47]. Recent studies report that exosomes from hypoxia-preconditioned adipose tissue MSCs significantly accelerate capillary network formation and fat graft survival by regulating VEGF/VEGFR signaling and through an increased expression of several proangiogenic growth factors [48]. Moreover, exosomes from hypoxia-treated MSCs are able to upregulate angiogenesis-related genes and activate protein kinase A (PKA) signaling, leading to an increase in VEGF expression [49].

Therapeutic effect of microvesicles depends on the source of parental cells and their bioactive content, and for potential clinical applications, extracellular vesicles obtained from MSCs of different tissue origins have been tested in different animal models as a cell-free therapeutic option (reviewed by [15, 25]).

The potential therapeutic application of extracellular vesicles in regenerative medicine or angiogenesis-related diseases is on focus in many experimental studies since they have the potential to deliver complex information to endothelial cells and to induce either pro- or antiangiogenic signaling (reviewed by Todorova et al. [50]). The angiogenic potential of extracellular vesicles originated from endothelial cells has been documented by the presence of bioactive factors (VEGF, PDGF, FGF-2, and RANTES) and microRNA with proangiogenic properties (miR-126, miR-216, miR-296, mir-31, and miR-150). However, the extracellular vesicles of endothelial cell origin play an important role in plasmin production, which affect the *in vitro* tube formation of endothelial progenitor cells in a dose-dependent manner, and showed that their high concentration inhibit angiogenesis. The antiangiogenic effect of extracellular vesicles is also induced by increased oxidative stress [50]. Microvesicles released from MSCs originated from bone marrow, umbilical cord, or adipose tissue exert proangiogenic effects mainly by transfer of bioactive factors produced by parental cells and by intercellular communication. The proangiogenic effect of microvesicles can be modulated by the culture conditions of parental MSCs. Modulating the culture condition of adipose tissue-derived MSCs with an endothelial differentiation medium resulted in increased proangiogenic properties of microvesicles carrying miR-31 [51].

The present study documented that microvesicles from immortalized MSC line (HATMSC1) of adipose tissue origin and from unipotential EPC line (HEPC-CB.1) have a distinct biomolecular composition that depends on cell source and culture conditions (hypoxia vs. normoxia). The microvesicle composition may exert different clinical effects, and this study proved that microvesicles from EPC line (HEPC-CB.1) transferred miRNA (miR-296) and growth factors (bFGF, VEGF, and IL-8) and proteins regulating angiogenesis (MCP-1 and TIMP) at a higher level than microvesicles from HATMSC1 cell line; however, the *in vitro* proangiogenic potential of microvesicles from both sources is similar.

Our results prove that microvesicles are important factors promoting the proangiogenic process, which is crucial for proper tissue regeneration. However, tissue regeneration is a complicated process, and microvesicles do not only affect angiogenesis. Further studies are needed to elucidate the role of intact microvesicles and the effect of microvesicle components on all biological processes during tissue regeneration.

## 5. Conclusions

The results presented in this study provide evidence that microvesicles from immortalized MSC line (HATMSC1) of adipose tissue origin promote angiogenesis as effectively as microvesicles derived from a unipotential EPC line (HEPC-CB.1). This observation clearly indicates that microvesicles isolated from both HEPC-CB.1 and HATMSC1 cell lines carry proteins and miRNAs that support and facilitate proangiogenic processes, which are important for proper tissue regeneration. Moreover, their effect on the proliferation of vessel endothelial cells reveals the role of microvesicles as a potential cell-free proangiogenic therapy in ischemic tissues that may overcome the disadvantages of current stem cell-based therapy.

## Data Availability

The data used to support the findings of this study are included within the article.

## References

[1] Klimczak A., Kozlowska U. (2016). Mesenchymal stromal cells and tissue-specific progenitor cells: their role in tissue homeostasis. *Stem Cells International*.

[2] Bieback K., Netsch P. (2016). Isolation, culture, and characterization of human umbilical cord blood-derived mesenchymal stromal cells. *Mesenchymal Stem Cells*.

[3] Kozlowska U., Krawczenko A., Futoma K. (2019). Similarities and differences between mesenchymal stem/progenitor cells derived from various human tissues. *World Journal of Stem Cells*.

[4] Orciani M., Di Primio R. (2013). Skin-derived mesenchymal stem cells: isolation, culture, and characterization. *Methods in Molecular Biology*.

[5] Ullah I., Subbarao R. B., Rho G. J. (2015). Human mesenchymal stem cells - current trends and future prospective. *Bioscience Reports*.

[6] Asahara T., Murohara T., Sullivan A. (1997). Isolation of putative progenitor endothelial cells for angiogenesis. *Science*.

[7] Fleissner F., Thum T. (2011). Critical role of the nitric oxide/reactive oxygen species balance in endothelial progenitor dysfunction. *Antioxidants & Redox Signaling*.

[8] Bian X., Ma K., Zhang C., Fu X. (2019). Therapeutic angiogenesis using stem cell-derived extracellular vesicles: an emerging approach for treatment of ischemic diseases. *Stem Cell Research & Therapy*.

[9] Sorg H., Tilkorn D. J., Hager S., Hauser J., Mirastschijski U. (2017). Skin wound healing: an update on the current knowledge and concepts. *European Surgical Research*.

[10] Vestweber D. (2015). How leukocytes cross the vascular endothelium. *Nature Reviews Immunology*.

[11] Than U. T. T., Guanzon D., Leavesley D., Parker T. (2017). Association of extracellular membrane vesicles with cutaneous wound healing. *International Journal of Molecular Sciences*.

[12] Velnar T., Bailey T., Smrkolj V. (2009). The wound healing process: an overview of the cellular and molecular mechanisms. *The Journal of International Medical Research*.

[13] Ferreira A. D. F., Gomes D. A. (2019). Stem cell extracellular vesicles in skin repair. *Bioengineering*.

[14] Harrell C. R., Fellabaum C., Jovicic N., Djonov V., Arsenijevic N., Volarevic V. (2019). Molecular mechanisms responsible for therapeutic potential of mesenchymal stem cell-derived secretome. *Cells*.

[15] Riazifar M., Pone E. J., Lotvall J., Zhao W. (2017). Stem cell extracellular vesicles: extended messages of regeneration. *Annual Review of Pharmacology and Toxicology*.

[16] Cabral J., Ryan A. E., Griffin M. D., Ritter T. (2018). Extracellular vesicles as modulators of wound healing. *Advanced Drug Delivery Reviews*.

[17] Trinh N. T., Yamashita T., Tu T. C. (2016). Microvesicles enhance the mobility of human diabetic adipose tissue-derived mesenchymal stem cells in vitro and improve wound healing in vivo. *Biochemical and Biophysical Research Communications*.

[18] Tu T. C., Yamashita T., Kato T. (2016). Microvesicles derived from Alde-Low EPCs support the wound healing capacity of AT-MSCs. *Biochemical and Biophysical Research Communications*.

[19] Kieda C., Paprocka M., Krawczenko A. (2009). New human microvascular endothelial cell lines with specific adhesion molecules phenotypes. *Endothelium*.

[20] Paprocka M., Krawczenko A., Dus D. (2011). CD133 positive progenitor endothelial cell lines from human cord blood. *Cytometry Part A*.

[21] Boukamp P., Petrussevska R. T., Breitkreutz D., Hornung J., Markham A., Fusenig N. E. (1988). Normal keratinization in a spontaneously immortalized aneuploid human keratinocyte cell line. *The Journal of Cell Biology*.

[22] Hurlin P. J., Maher V. M., McCormick J. J. (1989). Malignant transformation of human fibroblasts caused by expression of a transfected T24 HRAS oncogene. *Proceedings of the National Academy of Sciences of the United States of America*.

[23] Li X., Jiang C., Zhao J. (2016). Human endothelial progenitor cells-derived exosomes accelerate cutaneous wound healing in diabetic rats by promoting endothelial function. *Journal of Diabetes and its Complications*.

[24] Zhang J., Chen C., Hu B. (2016). Exosomes derived from human endothelial progenitor cells accelerate cutaneous wound healing by promoting angiogenesis through Erk1/2 signaling. *International Journal of Biological Sciences*.

[25] Rani S., Ryan A. E., Griffin M. D., Ritter T. (2015). Mesenchymal stem cell-derived extracellular vesicles: toward cell-free therapeutic applications. *Molecular Therapy*.

[26] Baran J., Baj-Krzyworzeka M., Weglarczyk K. (2010). Circulating tumour-derived microvesicles in plasma of gastric cancer patients. *Cancer Immunology, Immunotherapy*.

[27] Szatanek R., Baran J., Siedlar M., Baj-Krzyworzeka M. (2015). Isolation of extracellular vesicles: determining the correct approach (review). *International Journal of Molecular Medicine*.

[28] Witwer K. W., Buzas E. I., Bemis L. T. (2013). Standardization of sample collection, isolation and analysis methods in extracellular vesicle research. *Journal of Extracellular Vesicles*.

[29] Raposo G., Stoorvogel W. (2013). Extracellular vesicles: exosomes, microvesicles, and friends. *The Journal of Cell Biology*.

[30] Scuderi N., Ceccarelli S., Onesti M. G. (2013). Human adipose-derived stromal cells for cell-based therapies in the treatment of systemic sclerosis. *Cell Transplantation*.

[31] Kraskiewicz H., Paprocka M., Bielawska-Pohl A. (2020). Can supernatant from immortalized adipose tissue MSC replace cell therapy? An in vitro study in chronic wounds model. *Stem Cell Research & Therapy*.

[32] Majmundar A. J., Wong W. J., Simon M. C. (2010). Hypoxia-inducible factors and the response to hypoxic stress. *Molecular Cell*.

[33] Zhang H. C., Liu X. B., Huang S. (2012). Microvesicles derived from human umbilical cord mesenchymal stem cells stimulated by hypoxia promote angiogenesis both in vitro and in vivo. *Stem Cells and Development*.

[34] Gonzalez-King H., Garcia N. A., Ontoria-Oviedo I., Ciria M., Montero J. A., Sepulveda P. (2017). Hypoxia inducible factor-1*α* potentiates jagged 1-mediated angiogenesis by mesenchymal stem cell-derived exosomes. *Stem Cells*.

[35] Wang S., Olson E. N. (2009). AngiomiRs—key regulators of angiogenesis. *Current Opinion in Genetics & Development*.

[36] Anand S., Cheresh D. A. (2011). MicroRNA-mediated regulation of the angiogenic switch. *Current Opinion in Hematology*.

[37] Wurdinger T., Tannous B. A., Saydam O. (2008). miR-296 regulates growth factor receptor overexpression in angiogenic endothelial cells. *Cancer Cell*.

[38] Kovacic J. C., Moore J., Herbert A., Ma D., Boehm M., Graham R. M. (2008). Endothelial progenitor cells, angioblasts, and angiogenesis--old terms reconsidered from a current perspective. *Trends in Cardiovascular Medicine*.

[39] Sun L. L., Li W. D., Lei F. R., Li X. Q. (2018). The regulatory role of microRNAs in angiogenesis-related diseases. *Journal of Cellular and Molecular Medicine*.

[40] Ren S., Chen J., Duscher D. (2019). Microvesicles from human adipose stem cells promote wound healing by optimizing cellular functions via AKT and ERK signaling pathways. *Stem Cell Research & Therapy*.

[41] Khasawneh R. R., Al Sharie A. H., Abu-El Rub E., Serhan A. O., Obeidat H. N. (2019). Addressing the impact of different fetal bovine serum percentages on mesenchymal stem cells biological performance. *Molecular Biology Reports*.

[42] Bobis-Wozowicz S., Kmiotek K., Kania K. (2017). Diverse impact of xeno-free conditions on biological and regenerative properties of hUC-MSCs and their extracellular vesicles. *Journal of Molecular Medicine*.

[43] Huaitong X., Yuanyong F., Yueqin T., Peng Z., Wei S., Kai S. (2017). Microvesicles releasing by oral cancer cells enhance endothelial cell angiogenesis via Shh/RhoA signaling pathway. *Cancer Biology & Therapy*.

[44] Belik D., Tsang H., Wharton J., Howard L., Bernabeu C., Wojciak-Stothard B. (2016). Endothelium-derived microparticles from chronically thromboembolic pulmonary hypertensive patients facilitate endothelial angiogenesis. *Journal of Biomedical Science*.

[45] Cantaluppi V., Gatti S., Medica D. (2012). Microvesicles derived from endothelial progenitor cells protect the kidney from ischemia-reperfusion injury by microRNA-dependent reprogramming of resident renal cells. *Kidney International*.

[46] Deregibus M. C., Cantaluppi V., Calogero R. (2007). Endothelial progenitor cell derived microvesicles activate an angiogenic program in endothelial cells by a horizontal transfer of mRNA. *Blood*.

[47] Hong P., Yang H., Wu Y., Li K., Tang Z. (2019). The functions and clinical application potential of exosomes derived from adipose mesenchymal stem cells: a comprehensive review. *Stem Cell Research & Therapy*.

[48] Han Y., Ren J., Bai Y., Pei X., Han Y. (2019). Exosomes from hypoxia-treated human adipose-derived mesenchymal stem cells enhance angiogenesis through VEGF/VEGF-R. *The International Journal of Biochemistry & Cell Biology*.

[49] Xue C., Shen Y., Li X. (2018). Exosomes derived from hypoxia-treated human adipose mesenchymal stem cells enhance angiogenesis through the PKA signaling pathway. *Stem Cells and Development*.

[50] Todorova D., Simoncini S., Lacroix R., Sabatier F., Dignat-George F. (2017). Extracellular vesicles in angiogenesis. *Circulation Research*.

[51] Kang T., Jones T. M., Naddell C. (2016). Adipose-derived stem cells induce angiogenesis via microvesicle transport of miRNA-31. *Stem Cells Translational Medicine*.

